# Classification of foodborne pathogens using near infrared (NIR) laser scatter imaging system with multivariate calibration

**DOI:** 10.1038/srep09524

**Published:** 2015-04-10

**Authors:** Wenxiu Pan, Jiewen Zhao, Quansheng Chen

**Affiliations:** 1 School of Food and Biological Engineering, Jiangsu University, Zhenjiang 212013, China

## Abstract

An optical sensor system, namely NIR laser scatter imaging system, was developed for rapid and noninvasive classification of foodborne pathogens. This developed system was used for images acquisition. The current study is focused on exploring the potential of this system combined with multivariate calibrations in classifying three categories of popular bacteria. Initially, normalization and Zernike moments extraction were performed, and the resultant translation, scale and rotation invariances were applied as the characteristic variables for subsequent discriminant analysis. Both linear (LDA, KNN and PLSDA) and nonlinear (BPANN, SVM and OSELM) pattern recognition methods were employed comparatively for modeling, and optimized by cross validation. Experimental results showed that the performances of nonlinear tools were superior to those of linear tools, especially for OSELM model with 95% discrimination rate in the prediction set. The overall results showed that it is extremely feasible for rapid and noninvasive classifying foodborne pathogens using this developed system combined with appropriate multivariate calibration.

Food safety has emerged as an important global issue with international trade and public health implications, and foodborne outbreaks from microbial contamination, chemicals and toxins, take a major crisis on health. The World Health Organization (WHO) defines foodborne illnesses as diseases, usually either infectious or toxic in nature, caused by agents that enter the body through the ingestion of food^1^. Though the global incidence of foodborne disease is difficult to ascertain, it has been reported that in 2005 alone 1.8 million people died from diarrhoeal diseases and a great proportion of these cases can be attributed to biogenic contaminated food and drinking water^2^. Thus foodborne pathogens are the primary reason causing foodborne diseases, leading to an intense inspiration into the area of food pathogens detection which is the solution to the prevention and recognition of problems related to health and safety.

Conventional methods for the classification of microbial pathogenic agents mainly rely on specific microbiological and biochemical identification, among which, the culture and colony counting methods involve counting of bacteria, immunology based methods involve antigen-antibody interactions and the third polymerase chain reaction (PCR) method involves DNA analysis. However, although these methods can be sensitive, inexpensive and give both qualitative and quantitative information of the tested microorganisms, they are greatly restricted by assay time. While time and speed are of vital importance, they are not sufficient and should be accompanied by rapid prescreening methods, which would provide preliminary results immediately^3^,^4^. Besides, initial enrichment is needed in this process in order to classify pathogens which typically occur in low numbers in food.

Over the past few decades, there has been increasing attention on noninvasive light scattering technologies for studying on detecting bacterial cells^5^,^6^. Light scattering instruments such as surface plasmon resonance (SPR), flow cytometry and so on, have enabled the research of various biological molecules and ultimately have been a commercial success^7^,^8^. Besides, spectroscopic technologies for rapid identification of bacterial cells in suspension has become very popular^9^,^10^. However, there are challenges associated with bacteria in suspension, such as purity and homogeneity of cultures and arrangement of cells (usually in chains or clusters and few in individual) as the orientations of cells and distances between cells change with time. Whereas, the colony on a solid agar surface is more stable and its optical response could be modeled with scalar diffraction theory^11^. In recent years, an emerging technology of laser optical sensor, which was based on the concept that variations in refractive indices and size, relative to the arrangement of cells in bacterial colonies growing on a semi-solid agar surface will generate different light scatter patterns, has been studied combined with chemometrics, and achieved good performances^12^,^13^,^14^. However, visible laser was adopted in these researches mentioned above, which is susceptible to the external environment, so strict test requirements were demanded for the satisfied results^12^,^13^,^14^,^15^. The method like this has limitations in practical usage. NIR laser exhibits the properties of remarkably less biological damaging and deeper tissue penetration^16^; besides, it would not be absorbed by biological samples and induces no autofluorescence, and thus the signal-to-background ratio can be greatly improved^17^.

The main objective of this study was to develop a NIR laser scatter imaging system for classifying common foodborne pathogens. This system, as an efficient, noninvasive, reagent-less and user-friendly bio-sensing instrument, can be used for rapidly classifying bacteria. Specific procedures were outlined as follows: (1) a NIR laser scatter imaging system was developed to collect scattering images; (2) image normalization was performed by regular moment, and then the characteristic variables of Zernike moment invariances were extracted from the normalized images; (3) multivariate analysis tools such as linear discriminant analysis (LDA), K-nearest neighbor (KNN), partial least squares discriminant analysis (PLSDA), back-propagation artificial neural networks (BPANN), support vector machine (SVM), and online sequential extreme learning machine (OSELM) were applied comparatively for modeling; (4) the independent samples were used for model test.

## Results

**Figure 1:**
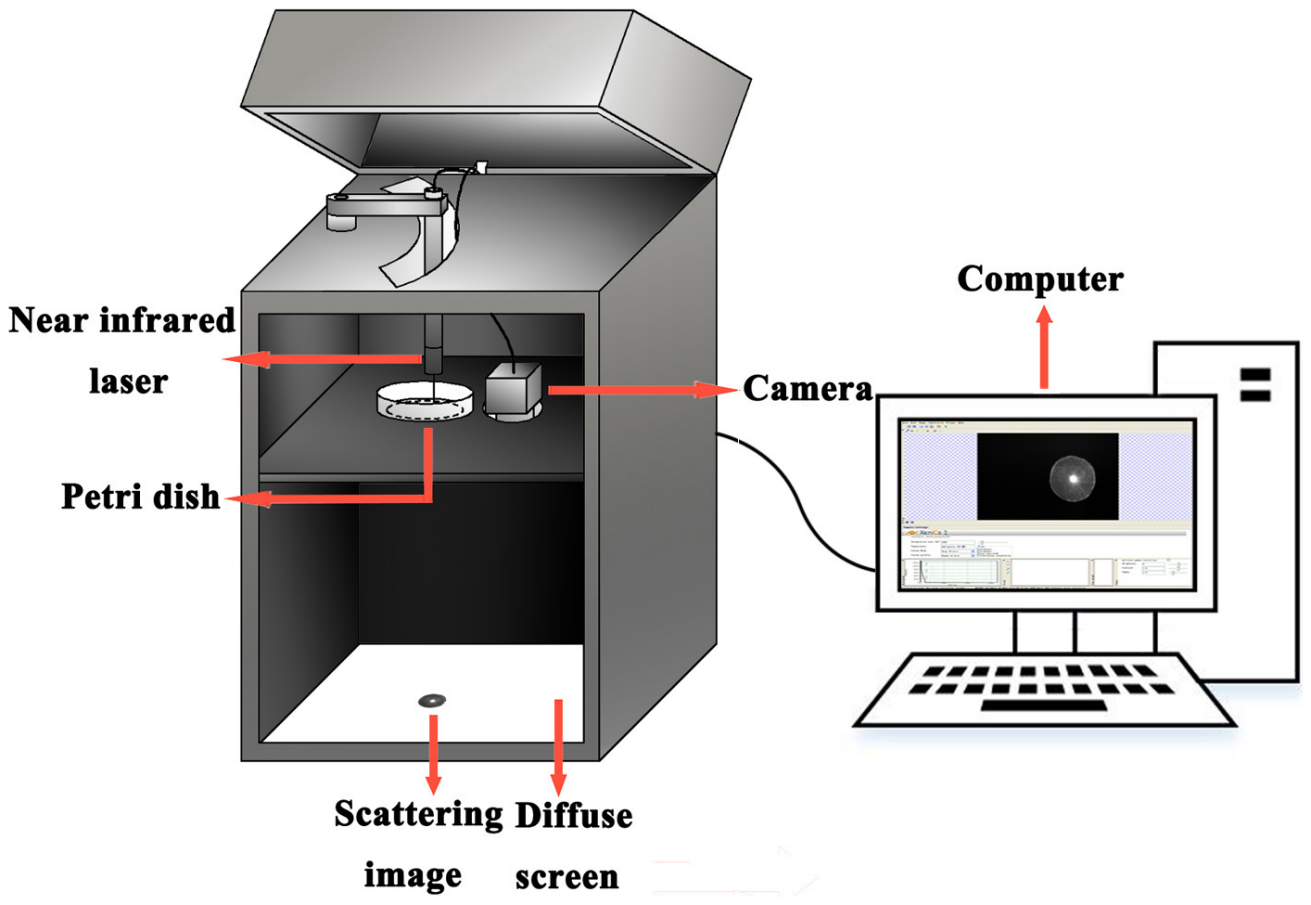
The sketch of NIR laser scatter imaging system.

**Figure 2:**
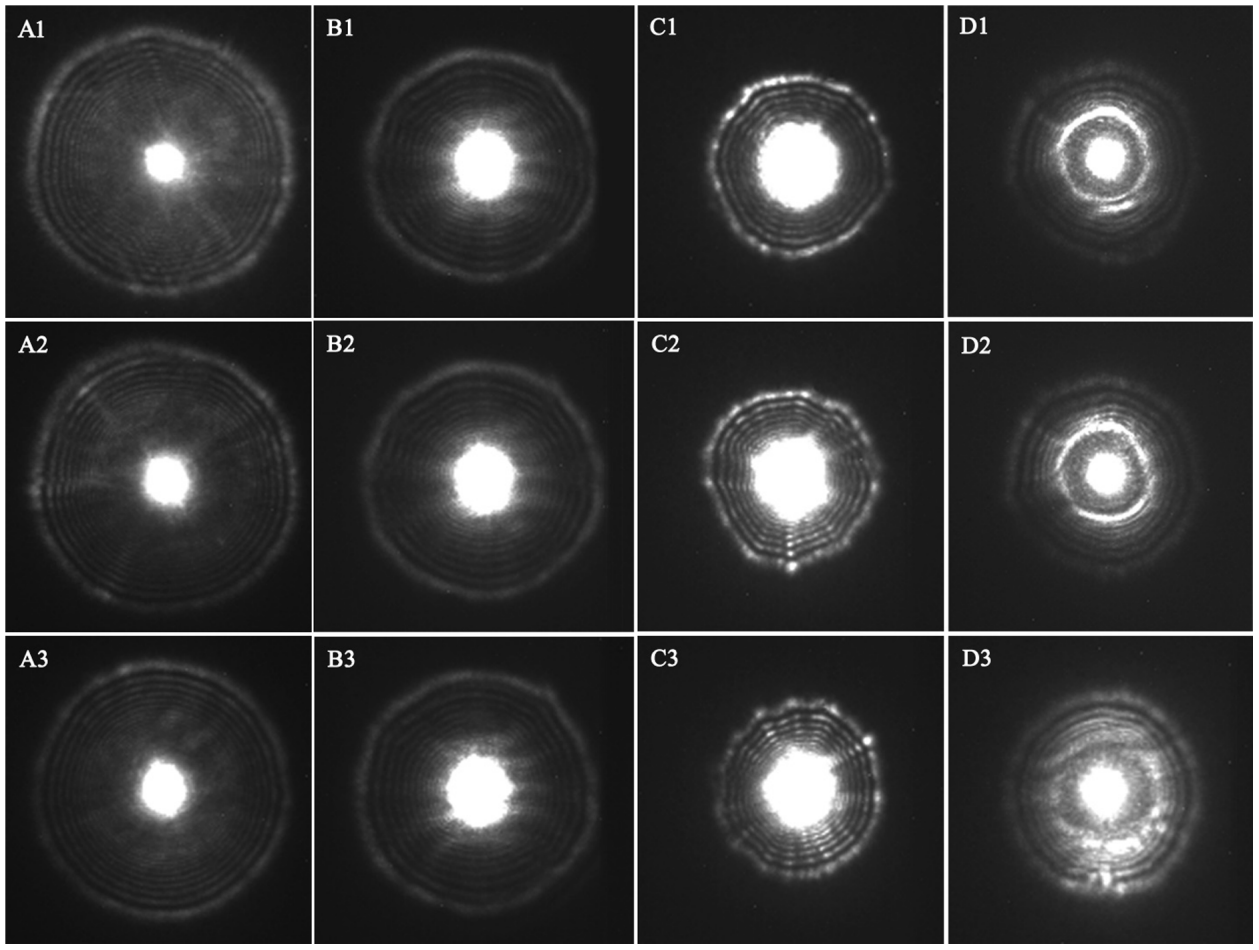
Representative original scattering images of the four categories of bacteria (Ai: *Escherichia coli*; Bi: *Staphylococcus aureus*; Ci: *Salmonella typhimurium*; Di: Mixed bacteria; each category presented three images, wherein, i = 1, 2, 3).

A bacterial colony was a characteristic dome-shaped structure formed by an exponential multiplication of a single cell on a nutrient agar surface. As can be seen in Figure 1, while the NIR laser lightened, a fraction of laser beam was fully transmitted through the bacterial colony and substrate, and another fraction was scattered (at relatively small angles to the transmitted beam). Thus the transmitted and scattered light formed the scattering image of the bacterial colony and projected on the diffuse screen, which was finally captured by the NIR camera; the former formed the bright center spot, and the latter formed a series of concentric rings and radial spokes as shown in Figure 2. Investigated from this figure, the size, sharpness and intensity of the center spot and the outer rings varied from strain to strain. Totally 120 scattering images of four categories were collected for the present study using the NIR laser scatter imaging system, and general results were described in detail as follows.

### LDA result

**Figure 3:**
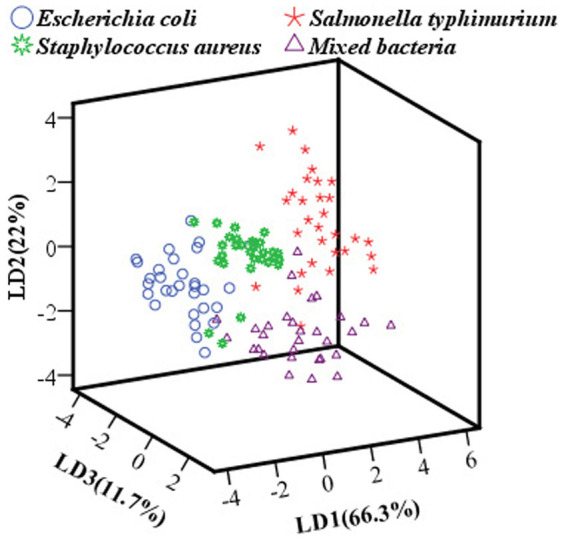
Score cluster plot with top three LDs of scattering images of four groups bacteria.

In this work, the result of LDA can be graphically visualized in Figure 3, and a clear cluster trend of scattering images of four groups was also displayed. LD1 explained 66.3% variance, LD2 explained 22% variance, and LD3 explained 11.7% variance, amounting to a total of 100% of the data variance. As investigated from this figure, it revealed that there are separations in four groups of scattering images, and all the images clustered well along the three LDs plane, except for a few images among these groups. The discrimination rates in calibration and prediction sets were 91.25% and 87.5%, respectively.

### KNN result

**Figure 4:**
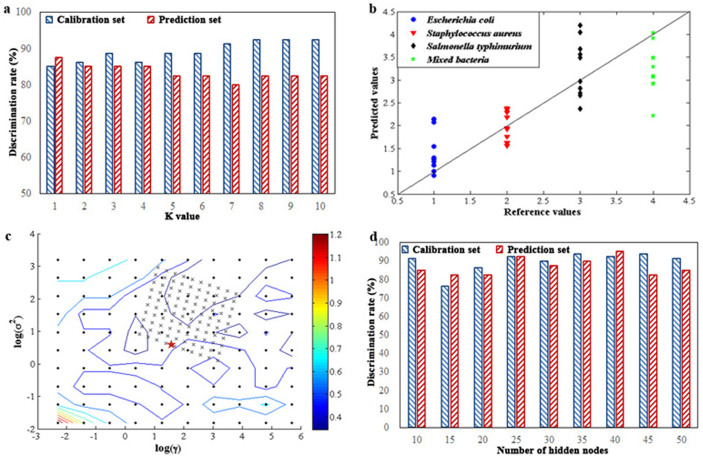
(a) Discrimination rates of KNN model according to different K values in the calibration and prediction sets; (b) prediction results of samples with different categories using PLSDA model; (c) contour plot of parameters optimization by cross-validation based on the calibration set; (d) OSELM model according to different numbers of hidden nodes in the calibration and prediction sets.

An appropriate value of K has a great influence on the discrimination rate of KNN model, which is determined by cross-validation with the lowest error in KNN model. Generally, parameter K is an odd number and less than 10, and the optimization result of KNN model by cross validation according to different K values in the calibration and prediction sets was shown in Figure 4a. As can be seen from this figure, the optimum KNN model was achieved when K = 1, resulting in discrimination rates of 85% in the calibration set and 87.5% in the prediction set.

### PLSDA result

In this case, scattering images of different categories (i.e., *Escherichia coli*, *Staphylococcus aureus*, *Salmonella typhimurium* and mixed bacteria) were assigned “1”, “2”, “3” and “4” as reference values, respectively. Figure 4b shows the prediction results of samples in the prediction set using PLSDA model. The diagonal line represents ideal results (reference values = prediction values), hence the closer the points are to the line, the better the model. As investigated from this figure, obviously, the overlap between different categories is so serious that it is hard to distinguish among them, with weak discrimination rates of 68.75% in the calibration set and 60% in the prediction set.

### BPANN result

Considering that the linear model may not provide a complete solution to such classification problem, BPANN as an efficient non-linear tool, was employed in contrast to the linear ones. Three layers (i.e., an input layer, a hidden layer and an output layer) of BPANN were arranged. Three latent variables of LD1, LD2 and LD3 extracted from LDA were used as the input of BPANN model, corresponding to 3 nodes in the hidden layer, other parameters were set as follows through substantial trials: the output layer was the classification labels of three categories of bacteria; scale function was the ‘tanh’ function; both the learning rate factor and momentum factor were all set to 0.1; the initial weights were set to 0.3; the maximum iteration times was set to 1000. Therefore, the final BPANN model was structured with a 3-3-1 topology, resulting in discrimination rates of 95% in the calibration set and 90% in the prediction set.

### SVM result

Generally, the structure of radial basis function (RBF) kernel function is the simplest and fastest for computation, thus it is chosen, and parameters including regularization parameter (γ) and kernel parameter (σ^2^) were optimized. The initial values of two parameters were pre-given, and optimization was based on the two pre-given initial values. In this case, the cost value was used to optimize SVM model and the optimum model was achieved according to the minimum of cost value. Figure 4c shows a contour map according to different cost values that are indicated by different colors. Results showed that the highest discrimination rates were obtained with 96.25% in the calibration set and 92.5% in the prediction set, corresponding to log(γ) = 1.3335 and log(σ^2^) = 0.3779, marked by a red asterisk in Figure 4c.

### OSELM result

Although BPANN and SVM are the most popular machine learning methods to classify patterns, they require an extensive training process and a complicated design procedure, hence OSELM was employed. In this study, the RBF activation function was chosen; the centers and widths of the nodes were randomly generated and fixed and then, based on this, the output weights were analytically determined. Considering that the number of hidden nodes has a significant effect on the performance of the model, it was optimized ranging from 10 to 50 and step 5, and determined according to the discrimination rate in the prediction set. Figure 4d exhibits the optimization result, and the optimal OSELM model can be achieved with discrimination rates of 92.5% in the calibration set and 95% in the prediction set when 40 hidden nodes were involved.

## Discussion

**Figure 5:**
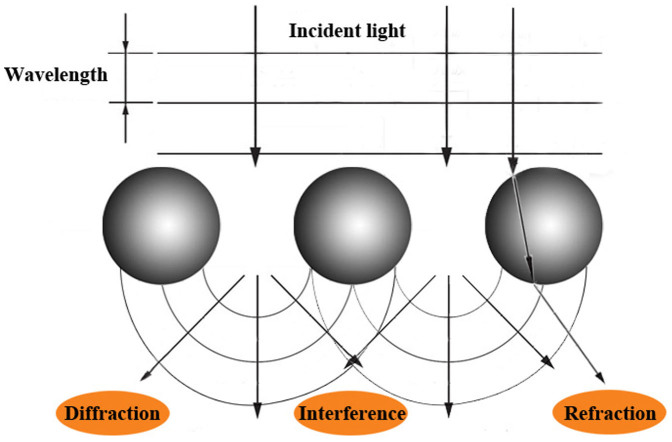
Theory of the phenomenon of light scattering.

Laser light scattering is generally a physical process where a beam of light is forced to deviate from its original straight trajectory and spread in all directions while through inhomogeneous medium. If the particles with the nanometer size are cluttered in the homogenous medium, such as emulsions, suspensions and so on, the original optical homogeneity will be destructed, leading to light diffraction and subwave interference^18^,^19^; besides, the refractive index of these particles is different from that of the homogeneous medium around, thus partial incident light will be refracted while cutting through the particles. Therefore, the appearances of diffraction, interference, refraction and so on, then ultimately construct the phenomenon of light scattering, as displayed in Figure 5.

When bacteria dilutions were evenly distributed on the surface of solid culture medium, bacteria grew and metabolized at the consumption of culture medium. After 18 to 24 h incubation, bacterial colonies formed and culture media was consumed, which would lead to the change of media compositions; a bacterial colony is composed of substantial bacterial cells, which is coincident with the conditions of light scattering. Hence, it is natural to see the scattering images in our self-assembling equipment as presented in Figure 2, which exhibited their specific textures according to their internal microstructures and compositions. In other words, these scattering images captured on the diffuse screen can also be considered for plane wave and Gaussian beam propagation^20^. Moreover, physical properties including refractive index, size, shape of bacterium, and biochemical compositions of bacteria and the remained culture media, are the main factors affecting the intensity distribution and polarization regulation of the scattered light. Therefore, scattering images of different categories of bacterial colonies differ from each other in terms of the size, sharpness and intensity of the center spot and the outer rings from visual analysis.

In order to maintain consistent among bacterial colonies of different plates, normalization was performed on the original images utilizing regular moments to obtain translation and scale invariances. Then Zernike moments of normalized images were extracted, thus translation, scale and rotation invariances were obtained as the characteristic variables for subsequent discriminant analysis, which are scalar values that do not change under affine transformations. A stepwise selection based on the value of the Wilks' Lambda (stepwise LDA) was first performed, and the cluster trend was presented in Figure 3. The contributions of the top three LDs were 100% for the total variances in the raw data; however, partial overlapping in the cluster trends was observed, especially between the three individual bacteria and the mixed bacteria, which may be explained as that the mixed bacterial colony was comprised of three bacterial cells, producing similar microstructures and biological compositions to the individuals. Therefore, in order to achieve a good performance in the classification of different categories of bacteria using the developed NIR laser scatter imaging system, six representative pattern recognition tools including linear (LDA, KNN and PLSDA) and nonlinear (BPANN, SVM and OSELM) tools, were systematically studied subsequent to general clustering and overall results were exhibited in Table 1. Investigated from Table 1, the results show that it is feasible and reliable to classify different categories of bacteria using this developed NIR laser scatter imaging system, and to some extent, nonlinear discrimination tools were superior to the linear ones, especially for the OSELM model. The main reasons could be summarized as follows.

Generally, LDA, KNN and PLSDA as the most commonly used linear discrimination methods, have presented their great potential in qualitative analysis^21^,^22^, thus they were chosen to apply in this study and all of them achieved good performances except for PLSDA model; this could be interpreted as follows: algorithms of LDA and KNN were used as soft classifiers where it is desired to estimate the probability that the correct identification is category, but PLSDA was used as hard classifier where the probabilities were not of primary interest, for instance, in cases where the object is easily classifiable by a human^23^. However, the correlation between the NIR laser scattering images and properties of bacterial colonies could incline to nonlinearity, whereas linear discrimination tools may not provide a complete solution to such nonlinear case in this work; moreover, nonlinear approaches are stronger than linear approaches in terms of self-learning and self-adjust^22^. Hence, BPANN, SVM and OSELM were chosen and attempted in this work, and also they both acquired better results, obviously superior to the linear approaches, which confirmed the nonlinear relationship between the images and biological properties.

Investigated between BPANN and SVM models, traditional BPANN is based on the empirical risk minimization (ERM) principle, which suffers difficulties with generalization producing model that can over-fit the data. On the contrary, the foundation of SVM embodies the structural risk minimization (SRM) principle, which has shown to be superior to the ERM principle, and the reason is that SRM minimizes an upper bound on the expected risk, as opposed to ERM that minimizes the error on the training data. Hence, SVM embodies better generalization in its theory, generating a better result than BPANN. Nevertheless, SVM also has its own deficiencies in terms of generalization performance and learning speed. Therefore, a sequential learning algorithm referred to as OSELM that can handle both additive and RBF nodes in a unified framework was introduced. Unlike other sequential learning algorithms which have many control parameters to be tuned, OSELM only requires the number of hidden nodes to be specified^24^. As seen in Table 1, the discrimination rate of the model using OSELM algorithm was improved a lot, producing better generalization performance at a significantly fast learning speed, which is of great significance to be applied in practical classification. Furthermore, the discrimination rate of the best model was still less than 100%, which may be explained as follows. On the one hand, the main part of error stemmed from environmental conditions and personal operation. On the other hand, the other part of error derived from inadequate or redundant variables, which can be interpreted as: Zernike moments were extracted as the characteristic variables, wherein, low order moments are usually utilized to describe the overall shape of an image object, and high order moments are applied to describe the details of an image object.

In conclusion, a NIR laser scatter imaging system was developed to obtain scattering images of bacterial colonies for the first time, which is noninvasive, cost-effective and environmentally friendly, providing rapid, reproducible results with minimal sample preparation. Combined with chemometrics, this system has a high potential in classification of different categories of bacteria; besides, results show that OSELM algorithm is extremely suitable for classifying different categories of bacteria. In the future, this system could be used to establish a database of scattering images for different bacteria, and then a huge discrimination model could be developed using an appropriate algorithm, which provides a promising tool for rapid and noninvasive classification of foodborne pathogens with high reproducibility. Also it has a very promising application prospect in food-processing industries and regulatory agencies for foodborne pathogens classification.

## Methods

### NIR laser scatter imaging system

The NIR laser scatter imaging system was developed and used for images acquisition of bacterial colonies. Figure 1 presents the sketch of this system, which was mainly comprised of four parts: (1) a 1-mW power NIR laser of 980 nm; (2) a NIR camera with spatial resolution of 320 × 256 pixels, ranging from 900 nm to 1700 nm; (3) a diffuse screen; and (4) a computer. The distance from the NIR laser to the petri dish, and the distance from the shelf holding petri dish and camera to the diffuse screen in the bottom, were maintained constant during whole images collection, and determined according to the individual experimental conditions. In this work, the former distance was 30 mm, and the latter distance was 285 mm. A collimated beam of light generated by NIR laser was 5 mm in diameter, and directed through the center of the bacterial colony and the substrate of bacterial agar medium, which finally projected onto the diffuse screen, and were collected as scattering images by NIR camera.

### Bacterial colony preparation

Three common foodborne pathogens such as *Escherichia coli* (ATCC 25922), *Staphylococcus aureus* (ATCC 25923) and *Salmonella typhimurium* (ATCC 14028) were used in this study. Considering the interaction influence of these bacteria in food, a mixture of three bacteria was regarded as the fourth category. The bacterial cultures were gradiently diluted in sterile water, so that the dilutions would produce about 50 to 80 colonies per plate. The dilutions were evenly distributed on the surface of solid culture medium and then incubated at 37°C for 18 to 24 h or until the colony reached 1 to 2 mm in diameter.

### Image acquisition and processing

Each category of bacteria was incubated for 5 plates, and thirty scattering images of each category were collected uniformly from these plates when integration time was set to 2400 ms, totally containing 120 scattering images. Figure 2 displays the original scattering images of four categories of bacteria.

In order to eliminate the effects of inconsistency among bacterial colonies of different plates, original images were subjected to a normalization process utilizing regular moments to obtain translation and scale invariances initially^25^. Regular moments are defined in the following equation:
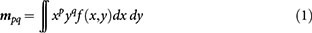
Where ***m****_pq_* is the (*p* + *q*)th order moment of the continuous image function *f* (*x*, *y*).

Translation invariancy is achieved by transforming the image into a new one whose first order moments, ***m***_01_ and ***m***_10_, are both equal to zero. This is done by transforming the original *f* (*x*, *y*) image into another one which is 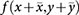, where 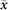 and 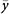 are the centroid location of the original image computed from 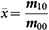, 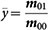.

Scale invariancy is accomplished by enlarging or reducing each shape such that its zeroth order moment ***m***_00_ is set equal to a predetermined value *β*. Let *f* (*x*/*a*, *y*/*a*) represent a scaled version of the image function *f* (*x*, *y*), and the scale factor *a* is set equal to 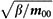.

**Figure 6:**
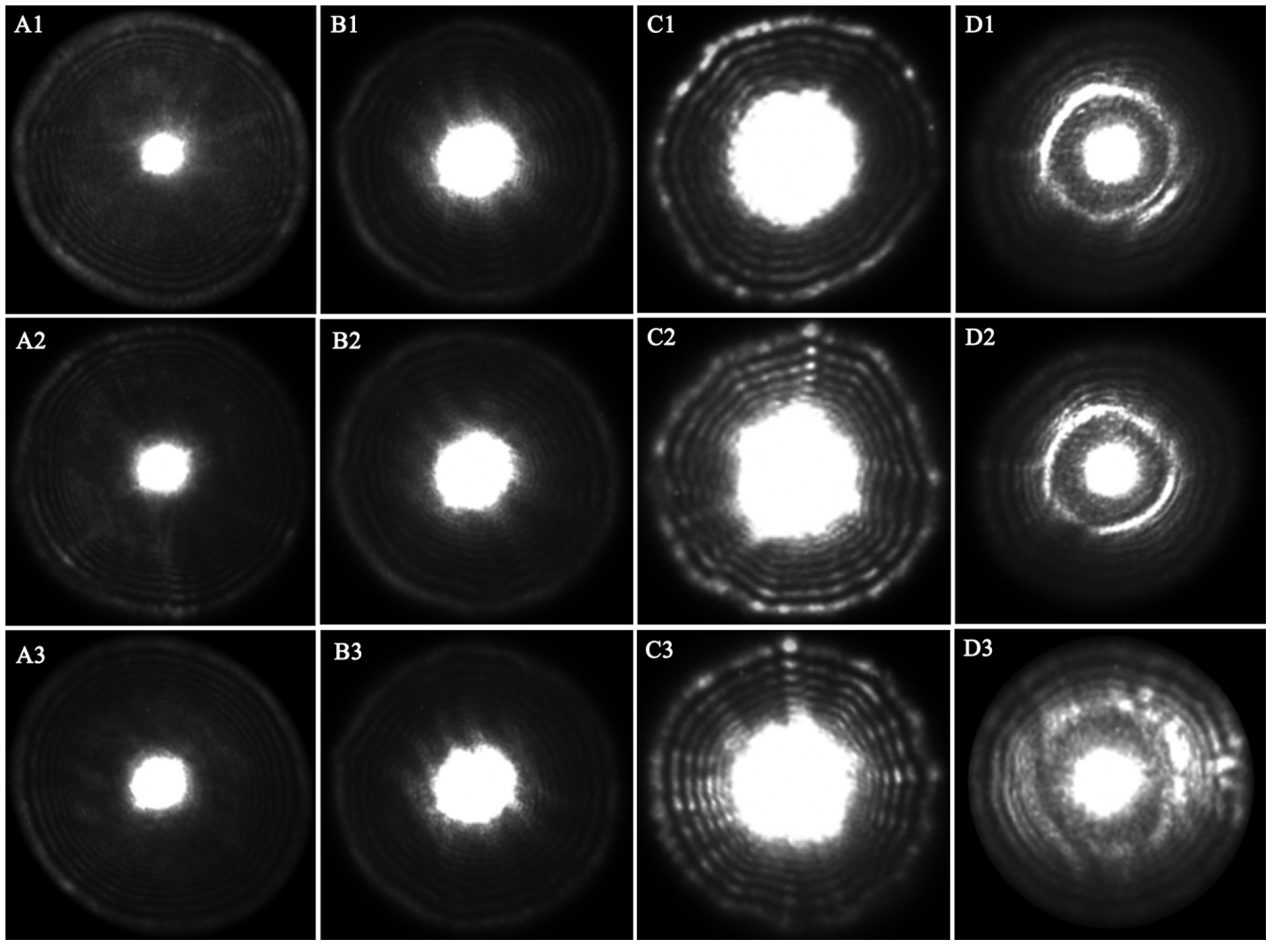
Representative normalized scattering images with translation and scale invariances of the four categories of bacteria (Ai: *Escherichia coli*; Bi: *Staphylococcus aureus*; Ci: *Salmonella typhimurium*; Di: Mixed bacteria; each category presented three images, wherein, i = 1, 2, 3).

In summary, an image function *f* (*x*, *y*) can be normalized with respect to scale and translation by transforming it into *g* (*x*, *y*) with *β* = 800, where
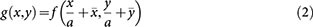
Figure 6 displays the normalized scattering images with translation and scale invariances of the original images.

### Characteristic variables extraction

Characteristic variables represent scalar properties of the objects, which plays an important role in character recognition. In many cases, a small number of characteristic variables are not sufficient for capturing enough information of an object, especially for some biological images. Characteristic variables like area, perimeter, compactness, and Fourier descriptors are too simplistic with their limitations such as requirement of clear and closed boundaries, and therefore more complex characteristic variables such as geometric and Zernike moment invariances find usage ranging from character and face recognition in computer vision to image registration^26^,^27^,^28^.

Subsequent to normalization, images with translation and scale invariances were obtained. For rotational invariance, Zernike moment invariances based on orthogonal polynomials, was first introduced by Teague^29^, with the property that it is straightforward to invert and thus easy to generate moment equivalent images based on a set of moments.

Zernike introduced a set of complex polynomials which form a complete orthogonal set over the interior of the unit circle (*x*^2^ + *y*^2^ = 1), and Zernike moments are the projection of the image function onto these orthogonal basis functions^30^. The Zernike moment of order *n* with repetition *m* for a continuous image function *f* (*x*, *y*) that vanishes outside the unit circle is
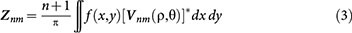
Where, *n* is positive integer or zero; *m* is positive and negative integer subjects to constraints *n* − |*m*| even, |*m*| ≤ *n*; *ρ* is the length of vector from origin to (*x*, *y*) pixel; *θ* is the angle between vector *ρ* and *x* axis in counterclockwise direction. Those pixels falling outside the unit circle are not used in the computation, thus note that 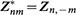.

It has been investigated that Zernike moments as the orthogonal moments are better than other types of moments in terms of information redundancy and image representation, and higher order moments are more sensitive to noise^31^. In this work, Zernike moment invariances of up to 20th order were calculated, and the resultant 90 characteristic variables including both low frequency shape information and high frequency details information, were used as inputs for the subsequent analysis procedure.

### Multivariate analysis

After characteristic variables extraction, it is necessary to select an appropriate multivariate analysis method for discriminating different categories of bacterial colonies. A stepwise selection based on the value of the Wilks' Lambda (stepwise LDA) was first performed on the 90 characteristic variables by reducing the dimension of the data matrix and compressing the information into interpretable variables, called latent variables. Then both linear and nonlinear discrimination tools, such as LDA, KNN, PLSDA, BPANN, SVM and OSELM, were applied comparatively using the latent variables in this work to develop discrimination models. The performances of these six models were evaluated according to their discrimination rates in the prediction set. All data analysis techniques were carried out in SPSS Version 16. 0. Lnk and Matlab Version 7.11.0 (Mathworks, Natick, USA) on Windows 7.

### (1) Theory of LDA

LDA is a supervised method utilized for classification purposes^32^, which maximizes the variance between categories and minimizes the variance within categories^33^. The latent variables obtained in LDA is a linear combination of the original variables; being *k* classes, *k* − 1 latent variables can be determined; the first LDA function is called LD1, the second is called LD2, and so on^34^. Therefore, the two or three dimensional representation of the topmost two or three LDs will be obtained to show the cluster trends of samples. More importantly, the topmost two or three LDs can be used as the input of the multivariate models.

After the initial cluster trends, the samples were divided into two sets, namely calibration set and prediction set. The calibration set was made up of 80 samples, which were used to develop the model, whereas, the remaining 40 samples in the prediction were used to validate the developed model.

### (2) Theory of KNN

The KNN classifier, first introduced by Fix and Hodges^35^, is a machine learning technique based on linear supervised pattern recognition. The class of an unknown observation is predicted by finding the K observations in calibration set that are closest in distance to this new observation and by choosing the class to which most of the K observations belong (majority voting)^36^.

### (3) Theory of PLSDA

PLSDA is a classification method on account of partial least squares (PLS) regression where the response variable is a categorical one (replaced by the set of dummy variables describing the categories) expressing the class membership of the statistical units^37^. The PLSDA model was developed by assigning the reference value (dummy variable) for each sample. The samples accompanied with a predicted value of ±0.5 as a cut-off were all considered to be correctly classified by the model, which was similar to those reported by others.

### (4) Theory of BPANN

Artificial neural network (ANN) was basically designed to mimic the biological nervous system that is capable of self-learning on samples^38^. Wherein, BPANN is the most classical feed-forward multi-layer networks based on an algorithm that corrects the weights within each layer in proportion to the error obtained from the previous layer, which is made up of neurons arranged in layers (an input layer, one or more hidden layers and an output layer) as the connection (weights) indirect from input to output^39^.

### (5) Theory of SVM

SVM developed by Cortes and Vapnik^40^, is a classifier used for performing classification tasks, which functions by constructing hyperplanes in a multi-dimensional space that separates cases of different class labels. It is a transformational tool that converts data from a low dimension input space to a high dimension feature space, which is implemented by a kernel function^41^.

### (6) Theory of OSELM

Extreme learning machine (ELM), as a new fast learning algorithm, proposed by Huang et al.^42^,^43^, has been developed for single hidden layer feed forward networks (SLFN). OSELM originates from the batch learning ELM, is a versatile sequential learning algorithm that can learn the training data not only one-by-one but also chunk-by-chunk (with fixed or varying length) and discard the data for which the training has already been done^44^.

## Figures and Tables

**Table 1:** Comparison of different multivariate analysis methods in the classification of different categories of bacteria

Methods	Calibration set		Prediction set	
	Samples	Discrimination results	Samples	Discrimination results
LDA	80	91.25%	40	87.5%
KNN	80	85%	40	87.5%
PLSDA	80	68.75%	40	60%
BPANN	80	95%	40	90%
SVM	80	96.25%	40	92.5%
OSELM	80	92.5%	40	95%
